# Antidepressants in the treatment of depression/depressive symptoms in cancer patients: a systematic review and meta-analysis

**DOI:** 10.1186/1471-244X-13-140

**Published:** 2013-05-16

**Authors:** Zacharias G Laoutidis, Klaus Mathiak

**Affiliations:** 1 Department of Psychiatry, Psychotherapy and Psychosomatics, RWTH University of Aachen, Bergische Landstrasse 2, 40629 Düsseldorf, Germany; 2 Department of Psychiatry, University of Düsseldorf, Bergische Landstrasse 2, 40629 Düsseldorf, Germany; 3 Jülich Aachen Research Alliance (JARA-BRAIN), Pauwelstrasse 30, Aachen, 52074, Germany; 4 Institute of Neuroscience and Medicine (INM-1), Forschungszentrum Jülich GmbH, Aachen, Germany

**Keywords:** Depression, Depressive symptoms, Cancer, Antidepressants

## Abstract

**Background:**

Over the past thirty years a number of studies have suggested that antidepressants can be effective in the treatment of depressive symptoms in patients with cancer. The aim of this paper was to review randomized controlled trials (RCTs) and to perform a meta-analysis in order to quantify their overall effect.

**Methods:**

Pubmed and the Cochrane libraries were searched for the time period between 1980 and 2010.

**Results:**

Nine RCTs were identified and reviewed. Six of them (with a total of 563 patients) fulfilled the criteria for meta-analysis, but exhibited an unclear risk for bias. The estimated effect size was 1.56 with 95% CI: 1.07- 2.28 (p= 0.021). There were no differences in discontinuation rates between antidepressants and placebo groups (RR= 0.86 with 95% CI 0.47- 1.56, p=0.62).

**Conclusions:**

This meta-analysis suggests that antidepressants can be effective in treating depressive symptoms beside clinical depression. When considering the risk of side effects and interactions and the heterogeneity among the mostly small studies, a general recommendation cannot be made until well-controlled studies are conducted.

## Background

The role of depression in physical illness has been recognized and addressed by many authors. Up to one third of physically ill patients attending hospital have depressive symptoms. The diseases with the highest prevalence of major depression have been reported as follows (from higher to lower incidence): inflammatory bowel disease, multiple sclerosis, epilepsy, asthma, back problems, cancer, COPD, migraine, rheumatic arthritis, stroke, Parkinson’s disease, diabetes mellitus, and heart disease [1]. Over the last several years, there has been a growing interest for the psychological aspects of cancer due to its severe impact on quality of life (QOL) [2,3]. Physical symptoms that are associated with both depression and cancer can be a confounding factor in the assessment of depression in this population [4]. Several studies evaluated the efficacy of psychological and pharmacological interventions in the treatment of depression. The psychopharmacological interventions and particularly the use of antidepressants are systematically reviewed here and meta-analytical methods are applied to quantify their overall effect.

Cancer is associated with depression and depressive symptoms. A meta-analysis by Mitchel et al. [5] included 94 studies and found that the pooled prevalence of major depression in palliative care settings and haemato-oncological settings was equal to 16.5% (95% CI: 13.1-20.3%) and 16.3% (95% CI: 13.4-19.5%), respectively. Restricting the analysis to standardized clinical assessment, Ng and colleagues [6] found a prevalence of 10.8% and also a substantial amount of heterogeneity. Even though these rates appear to be high, a consistent difference to a systematically matched sample from general population data is still controversial. The existing data suggest that “survivorship presents ongoing psychological challenges” [7].

There are two main confounding factors in the assessment of depression in cancer patients. First, the distinction between normal sadness or grief and symptoms indicating a depressive episode is not well-defined. Indeed, a phase of reduced mood or depression is considered part of healthy coping with grief (e.g. Kübler-Ross already in 1969 [8]). Further, such reaction patterns may recur as the disease progresses, by treatment failure, or by findings of metastases. Therefore, also time criteria may not capture the dynamics of disease progression.

A second confounder is the lack of specificity of the depressive symptoms. The ICD-10 and DSM-IV criteria for depressive episode include symptoms that are often present in patients with cancer as well, e.g. loss of appetite, low energy levels, or sleep disturbance. Therefore, the definition of depression in cancer patients and in physical illness is ambiguous [4]. It is suggested to identify the patients by their symptoms and not by a clinical syndrome because the ability to detect cases of depressive episode or disorder may be less important than the ability to detect depressive symptoms remediable to treatment [9]. Several approaches have been developed to solve the problem of diagnosing depression in cancer patients [10]. For instance, the substitutive approach suggests that all physical/somatic symptoms (change in appetite/weight, sleep disturbance, fatigue, loss of energy, diminished ability to think or concentrate) are replaced by non-somatic symptoms (tearfulness, depressed appearance, social withdrawal, decreased talkativeness, brooding, self-pity, pessimism, lack of reactivity, blunting) [11]. These are known as the Endicott criteria. Therefore, established criteria for depression may not be better suited to detect therapeutic indications in cancer patients than the presence of depressive symptoms.

Many guidelines for the treatment of cancer recommend that all cancer patients should be screened for depression, pain, and fatigue (e.g. by the National Institute of Health [12]). Multi-item scales are used for screening and diagnosing depression. Quantified results are used to specify the illness severity and to monitor the course of the disease. For detecting depression, ultra brief screening tools have been developed and proven to be reliable. For instance, Chochinov reported that the single question: “Are you depressed?” can be a reliable screening tool [13]. There is clear evidence that the systematic application of screening instruments reduces false negative findings, but the specificity and effects on outcome measures not sufficiently studied [14].

Depression seems to influence the prognosis and even the survival of cancer patients. For instance, Satin et al. conducted a meta-analysis with 27 studies on mortality in cancer patients and depression. A significant effect of depression on mortality was reported (RR: 1.25, P<0.001). The majority of studies measured the effect of depressive symptoms and only three of them included patients with clinical depression. A correlation with disease severity cannot be excluded [15]. In a large cohort study, patients who had recently received a cancer diagnosis had an increased risk for both suicide and death from cardiovascular causes as compared with controls [16]. Trials found that a decrease in severity of depressive symptoms is associated with a prolongation of survival in cancer patients [17,18]. However, these findings remain controversial as the majority of studies failed to replicate them [19-22].

## Treatment of depression in patients with cancer

The treatment of depression can mainly be divided into two categories: psychosocial and pharmacological interventions. A meta-analysis found that cognitive behavioral therapy has a positive effect on depression and quality of life in patients with cancer. In the same meta-analysis patient education had a positive influence on quality of life but not on depression [23]. A further meta-analysis of psychotherapeutic and psychopharmacological studies found allover positive effects on depression ratings [24]. Meta-analyses focusing on pharmacological treatment give a less consistent picture.

Previous reviews [6,25-28] underpinned the lack of evidence of the adequate effect of pharmacological interventions in the treatment of depression in cancer patients. However, the reviewed studies were heterogeneous as concerns the studied population (e.g. fatigue or pain as eligibility criterion and depression as secondary outcome) and the type of the drug applied (e.g. antidepressants, benzodiazepines, antipsychotics, psychostimulants, etc.). Only limited conclusions could be drawn from these reviews for the effectiveness of antidepressants in this population. A meta-analysis estimated the efficacy of antidepressants in palliative care (patients with cancer, HIV, COPD, etc.; [29]). The overall effect of antidepressants was significantly higher than the effect of placebo. However, only four of the twenty-five studies included cancer patients. A subgroup analysis was not performed for each subpopulation and thus no recommendation can be given for oncological patients. In another meta-analysis, antidepressants were found to be effective in the treatment of major depression with a co-morbid physical illness (RR= 1.42, P<0.0001). Again, only four RCTs were included that studied cancer patients with a diagnosis of major depression and no significant effect of antidepressants on response rates emerged in this subpopulation (RR=1.26, P=0.19) [30]. In contrast, Hart et al. [24] found a significant effect on depression ratings in the subgroup of four pharmacological studies which was not significantly different from the overall effect of the psychotherapeutic trials. However, this analysis included one placebo group twice and used Hedge’s g to quantify the results, which may bias statistics in the pharmacological subgroup. The authors discuss as a limitation that changes in questionnaire ratings may have limited clinical relevance. Depressiveness even without manifest diagnosis of depression may have adverse effects on prognosis and quality of life in cancer patients (see [14]) and, therefore, these patients should be included in intervention trials and subsequent meta-analyses. To overcome the limitations of the previous analyses, the present systematic review and meta-analysis focuses on the event of clinical relevant symptom changes in depressed or depressive patients with cancer.

## Methods

### Search strategy

The aim of the present study was to determine whether antidepressants are effective in the treatment of depression and depressive symptoms in patients with cancer. The inclusion criteria for the studies were:

1. Double-blind randomized-controlled trials (RCTs), which could be placebo-controlled or head-to-head trials. For the purposes of the meta-analysis only placebo-controlled trials were used.

2. The eligibility criteria of the studies were the presence of depression or depressive symptoms in patients with malignancy, i.e. impaired mood had to be diagnosed by clinical criteria or relevant depression rating scales.

3. The primary outcome of the studies was reduction in severity of depression or depressive symptoms.

4. The studies were published in English in the time between 01.01.1980 and 31.12.2010.

Antidepressants are often used for indications other than depression (e.g. fatigue, pain, hot flashes) in patients with malignancy. Thus, we excluded all studies, which had depression as a secondary outcome only.

We searched for studies in the electronic databases Pubmed and the Cochrane Library. We aimed towards higher sensitivity and lower precision in this first selection in order not to miss an appropriate study. In particular, we omitted any search term for therapy or treatment, which could reduce the search sensitivity. This approach is suggested by the “Cochrane Handbook for systematic Reviews of Interventions” (§6.4.4) [31]. Search terms were: “(depressive OR depression) AND (cancer OR tumor OR neoplasm OR lymphoma OR leukemia)”. The applied limits of the search were 1. articles should be published in the time between 01.01.1980 and 31.12.2010; 2. articles should be in English language; and 3. the search term appeared in the title or the abstract of the articles. We further searched through the reference lists of reviews and relative articles to identify any additional studies. Exploratory extensions of search terms (e.g. including ‘oncology’) did not yield additional studies.

### Article selection and review strategy

The selection of studies involved an initial screening of the title and the abstract in order to find studies, which were appropriate according to the inclusion criteria stated above. If it was not clear from the title or the abstract that the study should be rejected, the full text was obtained. The process was conducted independently by both authors in order to reduce the possibility of relevant articles being rejected.

The data were extracted independently by both authors. In case of disagreement, a clinician experienced in psycho-oncology and liaison psychiatry could be involved to mediate consensual decisions. A structured format was used as the one applied in the presentation of the single studies in the appendix. Dichotomous data were collected for the primary outcomes of this review (responders and non-responders to treatment). Secondary outcomes were the number of drop outs, the number of patients with adverse effects, and the quality of life.

### Statistical methods (meta-analysis)

A random effects model was applied in the meta-analysis because of the assumption that the true effect size was not the same in all studies. Indeed, there were marked differences between the studies regarding the type of cancer, the stage of cancer, the drug used in each study, and the design (intention to treat analysis or completers’ analysis). The risk ratio (RR with 95% confidence intervals) was preferred to odds ratio for the computation of the effect size because it has the advantage of being more intuitive [32]. Heterogeneity I^2^ was computed in order to assess the percentage of the overall variability attributed to the between studies variability.

The risk of bias in individual studies was evaluated using the Cochrane Collaboration’ s domain based tool which assesses allocation concealment, sequence generation, blinding, selective outcome reporting, and other sources of bias. Risk of publication bias was assessed using a funnel plot, i.e. a display of estimated study quality in terms of standard error and the reported effect size. The calculations were performed using standard formulas [32] in MicroSoft Excel (Excel 2003 Edition, MicroSoft, Redmond, CA). The statistical program “Comprehensive meta-Analysis” (2^nd^ version, Biostat, Englewood, NJ) was used to create forest and funnel plots.

## Results

### Search results

**Figure 1:**
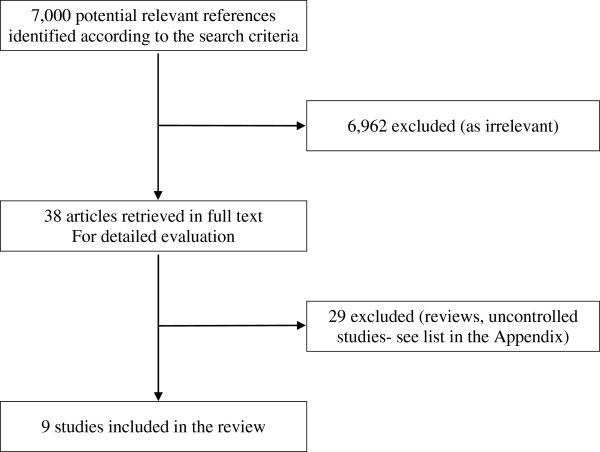
**Flow diagram of the study. **The electronic searches provided a total of 7000 references from MEDLINE and from the Cochrane Library. After the initial scanning of the abstracts a total of 38 reports remained. These reports were further screened and assessed for eligibility and 29 of them were rejected. The remaining 9 RCTs fulfilled the inclusion criteria for the review and six of them fulfilled the criteria for the meta-analysis.

The electronic searches yielded 5959 references from MEDLINE and 1041 references (clinical trials) from the Cochrane Library. After the initial scanning of the abstracts, a total of 38 reports were detected that may relate to drug trials using anti-depressants. Based on the full-text of these reports, 29 of them were rejected since they did not reported RCTs on anti-depressant treatment in depressive cancer patients Figure 1. From the remaining 9 RCTs, 3 studies were head-to-head trials, i.e. active drugs were compared with each other [33-35]. Thus in total 6 randomized placebo-controlled studies fulfilled the criteria for this meta-analysis [36-41]. Table 1 provides an overview of the reviewed studies. The complete list of the assessed trials is presented in Appendix A.

**Table 1:** Overview of the reviewed studies

**Author**	**Year**	**Drug**	**Study design**	**Participants (n)**	**Duration**	**Type of cancer/Stages**	**Evaluation**	**Results**
**A. Head-to-head trials**								
Holland	1998	Fluoxetine vs. Desipramine	Double blind RCT, ITT analysis, LOCF approach	n= 38	6 weeks	Breast cancer, Stages II, III, IV	HDRS Other: CGI, PGI, HAS, FLIC, MPAC, SF-36 HS	No significant difference between fluoxetine and desipramine.
Pezella	2001	Paroxetine vs. Amitriptyline	Double blind RCT, ITT analysis, LOCF approach	n= 179 177 received medication	8 weeks	Breast cancer, any stage	MADRS Other: CGI, FLIC, PGE	38/88 subjects in the drug group and 33/87 in the amitryptiline group were responders (p= 0.441)
Cancurtaran	2008	Mirtazapine vs. Imipramine vs. Control groupwithout medication	Double blind (for the participants of the two drug groups) RCT, completers’ analysis	n= 53	6 weeks	NR	SCID, HADS	Significant improvement in the mirtazapine group. No significant change in the other two groups.
**B. Placebo controlled studies**								
Costa	1985	Mianserin	Double blind RCT, ITT analysis, LOCF approach	n= 73	4 weeks	Gynecological cancer, stages II, III, IV	ZSDRS, HDRS, CGI-S	28/36 in the Mianserin group and 18/37 in the placebo group were responders (p< 0.025).
Van Heeringen	1996	Mianserin	Double blind RCT, ITT analysis, LOCF approach	n= 55	7 weeks	Breast cancer, stages I, II	HDRS	19/28 patients in the mianserin group and 10/27 in the placebo group were responders (p= 0.044).
Razavi	1996	Fluoxetine	Double blind RCT, completers’ analysis	n= 91	5 weeks	Any kind of cancer, any stage	HADS Other: MADRS, HAS, SCL-90R, SQOLI	18% in the fluoxetine group and 20% in the placebo group were responders (no significant difference)
Fisch	2003	Fluoxetine	Double blind RCT, Completers’ analysis	n= 163	12 weeks	Any type of cancer, advanced stage	TQSS, FACT-G, BZRDS	Results available for 129 patients. 31/64 patients in the fluoxetine group and 23/65 in the placebo group were responders (p= 0.12).
Musselmann	2006	Paroxetine vs Desipramine vs placebo	Double blind RCT, ITT analysis, LOCF approach	n= 35	6 weeks	Breast cancer, any stage	DSM-III-R- multiaxial evaluation HAD, HAS, CGI	5/13 in the paroxetine group, 5/11 in the desipramine group and 6/11 in the placebo group were responders (no statistical significance).
Navari	2007	Fluoxetine	Double blind RCT, completers’ analysis	n= 193	Six months	Breast cancer, stages I, II	TQSS, BZDRS, FACT-G	71/90 subjects in the fluoxetine group and 23/90 in the placebo group showed a significant (p< 0.01) improvement (p< 0.0005).

BZDRS: Brief Zung depression rating scale, CGI-S: Clinical global impression Scale for Severity of Illness, DSM: Diagnostic and statistical manual for mental disorders, FACT-G: Functional assessment of cancer therapy- global, FLIC: Functional living index- cancer, HAD: Hamilton Depression Scale, HADS: Hospital anxiety and depression scale, HAS: Hamilton anxiety scale, HDRS: Hamilton rating depression scale, ITT: Intention-to-treat, LOCF: last observation carried forward, MADRS: Montgomery and Asberg depression rating scale, MPAC: Memorial pain assessment card, NR: Not reported, PGE: Patients’ global evaluation, PGI: Patients’ global impression, RCT: Randomized controlled trial, SCID: Structured clinical interview for DSM, SF36-HS: Short Form 36 Health survey, SCL-90-R: Symptom Checklist 90, Revised, SQOLI: Spitzer Quality of Life Index, TQSS: Two questions screening survey, ZSRDS: Zung self-rating depression scale.

Previous reviews and meta-analyses exhibited a larger diversity of study designs [25-28]. For instance, we did not include 3 trials that had not depression or depressive symptoms as an eligibility criterion even though the primary outcome measure was improvement in depression/depressive symptoms [42-44]. Similarly, we did not include trials which tested the efficacy of antidepressants in preventing depressive symptoms in patients with cancer [45] or in patients with melanoma undergoing therapy with interferon [46]. Appendix D lists all the 38 trials, which were screened and the reasons for in- or excluding them.

### Review of RCTs in depression

A. Head-to-head trials

Holland et al. [33] studied 38 patients with depression and breast cancer. The selective serotonine reuptake inhibitor (SSRI) fluoxetine was not found to be superior to the tricyclic antidepressant (TCA) desipramine. Similarly, Pezella et al. [34] found no significant differences in efficacy between paroxetine (SSRI) und amitryptiline (TCA) in a sample of 185 patients with breast cancer. In contrast, the noradrenergic and specific serotonergic antidepressant (NaSSA) mirtazapine had a larger effect on depression than imipramine in a sample of 53 cancer patients with depression (Cancurtaran et al. 2008; [35]).

B. Placebo-controlled studies

Costa et al. [36] and van Heeringen et al. [37] compared the tetracyclic antidepressant mianserin with placebo in 73 and 55 patients with gynecological tumors, respectively. Both publications reported a significant effect on the observed depressive symptoms. In contrast, a trial by Razavi et al. [38] in 91 patients with various types of cancer did not reveal a significant difference between fluoxetine and placebo. Similarly, Fisch et al. [39] failed to demonstrate an advantage of fluoxetine over placebo in a sample of 163 patients with advanced cancer. A reevaluation of the results of the former study with the generalized estimating equation (GEE) method of regression suggested a significant effect of the verum as well. Navari et al. [40] found a significant effect of fluoxetine in 193 depressive patients with breast cancer. Finally, Musselmann et al. [41] could not document a drug effect in a trial with a small number of patients (n= 35) and 3 groups (paroxetine, desipramine, placebo). For the purposes of the current meta-analysis, we created a combined intervention group, which included the patients from both the paroxetine and the desipramine group, as recommended in the Cochrane handbook [31].

### Meta-analysis

#### Effect size

**Figure 2:**
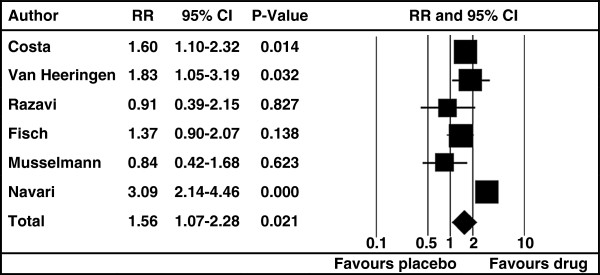
**Forest plot of RR with CI for all studies and overall. **The overall effect size in the analysis is RR=1.56 with 95%-CI: 1.07- 2.28 (p=0.021). This means that the effect of antidepressants in this population is significant better than the placebo effect. Four studies found a positive effect of the antidepressants on depressed cancer patients. In two studies the antidepressant was not better than the placebo. The 95%-CIs of these two studies were wider than the ones of the other four studies, which is indicative of low precision. RR: relative risk; CI: confidence intervals.

All studies defined a measure of response, i.e. what was considered a meaningful improvement of the depressive symptoms. The overall effect size in the analysis is RR=1.56 with 95%-CI: 1.07-2.28 (p= 0.021), i.e. under the antidepressants a therapeutic response (as defined in the considered studies) is about 50% more likely than in the placebo group (see Table 2). A graphical display of the relative strength of each study is presented in the forest plot (Figure 2). Four studies found a positive effect of the antidepressants on depressed cancer patients. In the other two studies no significant difference emerged but the 95% confidence intervals were wider than those of the four other studies (Figure 1). This can be considered as indicative of low precision in the trials with negative finding.

**Table 2:** Number of participants in both groups (drug and placebo) for each study

**Author**	**Year**	**Verum**	**Verum**		**Placebo**		**RR**
			**Participants**	**Responders**	**Participants**	**Responders**	
Costa	1985	Mianserin	36	28	37	18	1.60
Van Heeringen	1996	Mianserin	28	19	27	10	1.83
Razavi	1996	Fluoxetine	45	8	46	9	0.91
Fisch	2003	Fluoxetine	64	31	65	23	1.37
Musselmann	2006	Paroxetine	24	11	11	6	0.84
Navari	2008	Fluoxetine	90	71	90	23	3.09
			287	167	276	89	

#### Heterogeneity

The meta-analysis revealed a substantial heterogeneity I^2^= 71% with 95% CI: 54%-82%. For a substantial I^2^ (50-90%), the “Cochrane Handbook for systematic Reviews of Interventions” [31] recommends a reanalysis without the outlying studies as part of a sensitivity analysis. Indeed, the RR of the study by Navari et al. [40] is much higher than all other values (see Figure 2) and the analyses were repeated excluding this study. The effect of the antidepressants remained significantly better in comparison to placebo after excluding the outlying study (RR = 1.39, 95%-CI: 1.09- 1.77, p = 0.008) and the heterogeneity decreased to 10% (95%-CI 0-22%). Heterogeneity between 0% and 40% is considered to be of no importance [31]. This finding confirms that the high heterogeneity is most likely due to one outlier which however does not bias the finding.

#### Subgroup analysis

Four factors differentiating the studies were identified post-hoc. The selected predictors were: 1. depression vs. depressive symptoms as eligibility criteria; 2. analysis on an intention-to-treat (ITT) basis vs. completers’ analysis; 3. inclusion of all or only advanced cancer stages vs. inclusion of only early stages; and 4. the substance group SSRI vs. tetracyclic antidepressants (mianserin). In this post-hoc analysis, mianserin had significantly higher RR for responders than the SSRIs (RR_SSRI _= 1.16, RR_mianserin_ = 1.67, Z_difference _= -2.17, p = 0.03). In this latter subgroup comparison, only the paroxetine group from the study by Musselmann et al. was contrasted in order to make the intervention groups comparable. The other predictors did not significantly influence the RR for antidepressant effects. Appendix C provides the details of the subgroup analysis.

#### Adverse effects and dropouts

There was a substantial amount of missing data concerning the adverse effects in these studies. Only three studies reported the total number of patients with side effects. Four studies provided data about the number of drop outs because of side effects in each arm (Table 3). Visual inspection suggested no difference. However due to missing data, we did not perform a meta-analysis for the adverse effects. All but one studies provided information about the number of dropouts in each arm (Table 4). We performed a meta-analysis using relative risk ratios and found no significant difference between dropouts in the verum and placebo groups (mean RR = 0.86, 95%-CI 0.47- 1.56, p = 0.62).

**Table 3:** Number of patients with adverse events

**Author**	**Year**	**Drug**	**Verum**		**Placebo**	
			**Participants**	**A.E.**	**Participants**	**A.E.**
Costa	1985	Mianserin	36	17	37	11
Van Heeringen	1996	Mianserin	28	11	27	17
Razavi	1996	Fluoxetine	45	20	46	23
Fisch	2003	Fluoxetine	64	NR	65	NR
Musselmann	2006	Paroxetine	24	NR	11	NR
Navari	2008	Fluoxetine	90	NR	90	NR

A.E.: adverse event; NR: not reported.

**Table 4:** Dropouts

**Author**	**Year**	**Verum**		**Placebo**		**RR**
		**Participants**	**Dropouts**	**Participants**	**Dropouts**	
Costa	1985	36	7	37	15	0.48
Van Heeringen	1996	28	6	27	15	0.39
Razavi	1996	45	15	46	7	2.19
Fisch	2003	83	19	80	15	1.22
Musselmann	2006	24	10	11	5	0.92
		216	57	201	57	*0.86*

RR: relative risk.

#### Quality of life

Only three of the six studies included an outcome measure for quality of life. Razavi et al. [38] used the Spitzer Quality of Life Index (SQOLI). The increase in the SQOLI scores was significant in both the drug and the placebo group, but the difference between the two groups was not statistically significant. Fisch et al. [39] used the Functional Assessment of Cancer Therapy-General (FACT-G, version 3). There was no significant difference between the fluoxetine and the placebo group in the proportion of responders (six points change). Using the generalized estimating equations (GEE) method of regression (post-hoc), there was a significant improvement in the total FACT-G scores in the fluoxetine group compared with placebo. Navari et al. [40] also used the FACT-G scale. The number of patients who had a significant improvement in quality of life was statistically significantly higher in the fluoxetine group as compared to the placebo group.

#### Risk for bias and publication bias

**Figure 3:**
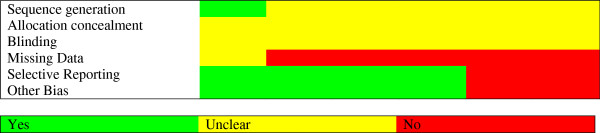
**Risk of bias graph. **The semaphore colors provide a visual impression of the quality of the study reports for meta-analysis; green: condition is fulfilled; yellow: condition is questionable and; red: condition is not fulfilled and risk of bias is present. The allover quality is unclear and indications for risk of bias can be derived. Therefore the meta-analysis cannot provide a high degree of level of evidence.

**Figure 4:**
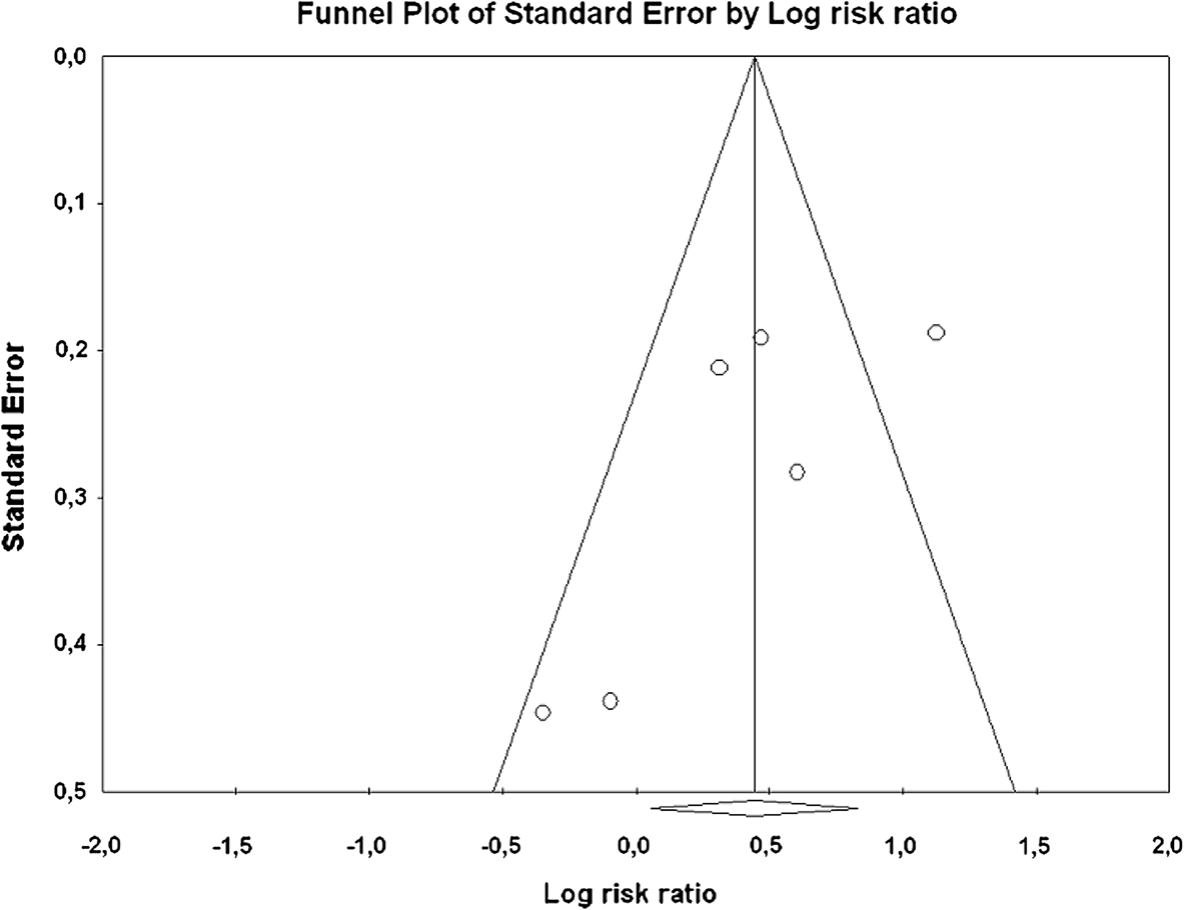
**Funnel plot. **The funnel plot reveals no gap on the left bottom size as an indicative for selective reporting of positive findings in studies with larger error and few participants. On the contrary there is a gap on the right size towards the bottom. Conceivably this is a random effect because of the small number of studies.

The risk of bias for each study can be determined by assessing the following six domains: 1. sequence generation, 2. allocation concealment, 3. blinding, 4. missing data, 5. selective outcome reporting, and 6. other sources of bias [31]. The group of studies was relative homogenous and the overall risk for bias could be described as “unclear” (Figure 3). The results for every single trial are presented in the Appendix B. The risk for publication bias (i.e. studies with small sample size are more likely not to be published if their effect is small to moderate) is assessed by means of the funnel plot, which displays the relationship between the sample size and the effect size of the studies. The standard error instead of the sample size is usually used in the Y axis. No indication for publication bias can be derived from the present funnel plot; in particular, there was no gap on the bottom left side, which would be indicative of unpublished studies with small to moderate effects (Figure 4).

## Discussion

This is the first systematic review with meta-analysis which focuses exclusively on the psychopharmacological treatment in cancer patients with depression. Treatment with SSRI or tetracyclic antidepressants was found to improve depressive symptoms more than placebo. The small number of studies and patients included, as well as the questionable risk of bias, however, points out the demand for well-conducted trials before general recommendations can be derived. The diagnosis and treatment of depression is of high importance in this population of patients because of the high risk for suicide [16], its impact on the quality of life [2,3], and its influence on anticancer treatment adherence and compliance [47]. Critical for an antidepressive treatment in this group of patients is a well-funded base of evidence since elevated risks of adverse effects and interactions must be expected (see *adverse effects and side effects* below). Interestingly in some cancer patients, subsyndromal depressive symptoms (with DSM-IV or ICD-10 diagnosis) may improve under the antidepressants as well. Direct comparisons between classical tricyclic antidepressants and SSRIs revealed no differences in two of the three reviewed head-to-head trials. The higher effect size of the tetracyclic agent mianserin in comparison to SSRIs – as seen in the subgroup analysis – must therefore be considered exploratory. At this point no specific recommendation concerning effectiveness for a substance class can be made.

Several studies and meta-analyses have reported on the effectiveness of antidepressants in patients with depression and physical illness. Van der Feltz-Cornelis et al. [48] showed in a meta-analysis that pharmacological interventions are effective in reducing depressive symptoms in patients with diabetes mellitus. Price et al. [49] found in another meta-analysis a significant effect of antidepressants in treating depression in patients with neurological disorders. There are RCTs which show a positive effect of SSRIs on depression in patients with asthma [50,51]. SSRIs were also found to be effective in the treatment of depression in patients with coronary artery disease [52]. Although the prevalence of depression in patients with inflammatory bowel disease (IBD) is high [1], there are no RCTs which assess the treatment of depression in this population [53].

### Depression and depressive symptoms

There is a trend towards identifying depressive symptoms instead of trying to define exact diagnoses of concrete depressive syndromes [9,10]. There are also many simple single- or two-item screening tools, which can detect depression with high specificity and sensitivity. As shown in the subgroup analysis, patients with depressive symptoms can benefit from the use of antidepressants exactly like the patients diagnosed with major depression. On the one hand, this conclusion is of significant clinical importance because it addresses a practical issue and can motivate physicians to screen for depression with simple and easy to use tools. On the other hand, it must be taken into consideration that there was only one study in one of the two compared groups in the subgroup analysis [39]. The authors of this study reported a better response in the patients who had a score higher than 4 in the TQSS, suggesting that a minimum of symptom severity may be required for the antidepressive action of antidepressants. Thus, the results of the subgroup analysis should be interpreted very carefully and not be misinterpreted as an excuse for an unreasonable use of antidepressants by physicians or for the limitation of the role of the consultation-liaison psychiatry in oncology.

### Side effects and interactions

When using an antidepressant, one should pay attention on possible side effects such as pro-emetic effects of SSRIs and anticholinergic effects of TCA. Nausea is a common adverse effect among cancer patients undergoing chemotherapy and can be worsened by SSRIs. Cognitive impairment or acute psychiatric conditions such as delirium can also get worse through the anticholinergic properties of TCAs. Adverse effects such as agranulocytosis with mianserin [54] should be taken into consideration in the treatment of cancer patients who receive chemotherapy.

There are also many interactions between antidepressants and drugs used in the treatment of cancer. The best studied interactions are these between SSRIs and tamoxifen (a Selective Estrogen Receptor Modulator or SERM), which is metabolized by CYP2D6 into its active form endoxifen [55]. Antidepressants such as paroxetine and duloxetine can inhibit the CYP2D6 cytochrome and thus the formation of the active metabolite endoxifen [56].

### Limitations

The heterogeneity between studies was substantial (I^2^= 71% with 95% CI: 54%-82%). As recommended in the Cochrane guidelines (see above), the meta-analysis was recalculated excluding an outlying value (as part of a sensitivity analysis). The efficiency remained significant even after removing an extreme high value (M*= 1.39, p= 0.008). The heterogeneity fell to 10% which is considered to be of no importance. Thus, heterogeneity affects psychopharmacological studies in cancer patients. Nevertheless, the overall therapeutic effects seem to be consistent across studies.

The reliability of these results is limited by the small number of randomized controlled studies. A larger number of studies are needed to get safe conclusions. The small number of studies and participants can be attributed among other reasons to the preference of the patients and the clinicians for non-blinded treatment, as reported by Musselmann et al. [41]. This may explain the large number of open label studies found in the search in the databases (which are not presented here). Another factor which limits the validity of our results is the quality of the studies. The average risk for bias in these studies could be described as “unclear.” As shown in the risk for bias graph (Figure 3), the group of studies was relatively homogenous as regards to this issue. Other limitations of this meta-analysis are the use of different depression rating scales and the different response criteria used by the authors.

## Conclusions

Considering the high prevalence of depression and its impact on mortality and quality of life in cancer patients, it is a matter of concern that only a few trials assessing antidepressant efficacy are available. Given this limitation, we found that antidepressants are effective in the treatment of depression or depressive symptoms in patients with cancer. A minimum of depressive symptoms’ severity may be required for the patients to benefit from the use of antidepressants. Though a larger effect size of mianserin in comparison to SSRIs in the subgroup analysis has been shown, no recommendation can be made for one antidepressant type over another. A quantification of tolerability, as ascertained by comparing the number of patients with adverse effects, was not possible because of the missing data. The number of drop outs did not differ significant between the intervention and the control group.

There are difficulties in defining the diagnosis of clinical depression in cancer patients. Symptoms such as fatigue, sadness, worry, and pain are reported by depressed patients as well as in patients with advanced disease. Practical issues such as the ability of physicians to recognize patients with depression should also be considered. Reliable single- or two-item questionnaires have been developed for this purpose. The detection of depressive symptoms might be more important than the exact diagnosis of clinical depression. The current meta-analysis suggests further that antidepressants could be effective in treating sub-clinical depression. However, studies with larger samples are needed in order to verify such conclusion before general clinical recommendations can be derived.

## Appendixes

### Appendix A

#### Listing of the studies

After scanning 7,000 references and screening 38 articles that seemed appropriate 9 trials could be identified to fulfill the study inclusion criteria. They were further categorized into 2 clusters:

A. **Head-to-head trials**

Three RCTs compared two antidepressants with each other (head-to-head trials). These studies did not include a placebo control group.

Holland et al., 1998

*Number of patients:* 40 patients were recruited and 38 ones of these were randomized (21 patients to the fluoxetine and 17 to the desipramine group).

*Type of cancer/Stages:* Breast cancer. Stages: II, III, IV.

*Duration:* six weeks

*Evaluation tools:* the Hamilton Depression Rating Scale (17 item HAM-D), the Clinical and Patient’s Global Impression scales (CGI and PGI), the Hamilton Anxiety Rating scale (HAM-A), the Functional Living Index for Cancer (FLIC), the Memorial Pain Assessment Card (MPAC) and the SF-36 Health Survey.

*Inclusion criteria:* Major depressive episode or adjustment disorder with depressed mood and scores over 14 on the first 17 items of HAM-D.

*Response criteria:* a statistically significant baseline to endpoint change in HAM-D.

*Design:* double blind RCT with ITT analysis, LOCF approach.

*Results:* There was a significant improvement in the HAM-D scores in both group as evidenced by baseline-to-endpoint changes (p<0.001). ANOVA showed no significant difference between fluoxetine and desipramine. Similar improvement was also observed in the HAM-A. There were no significant differences in the dropout rates in the fluoxetine and desipramine group (28.6% and 41.2%, respectively). The only adverse effect revealing a significance difference was dry mouth reported by 14 (66.6%) patients in the fluoxetine group and 4 (23.5%) patients in the desipramine group.

Pezzela et al., 2001

*Number of patients:* 179 patients were randomized into either the paroxetine group (n= 89) or the amitiptyline group (n=90). 88 patients from the first and 87 from the second group received medication and were included in the ITT analysis.

*Type of cancer/Stages:* Breast cancer, any stage.

*Duration:* eight weeks

*Evaluation tools:* Montgomery and Asberg Depression Rating Scale (MADRS), the Clinical Global Impression (CGI), the functional living index: cancer (FLIC) and the patient’s global evaluation (PGE).

*Inclusion criteria:* The patients should fulfill the ICD-10 criteria for depressive episode and have a minimum score of 16 in the MADRS.

*Response criteria:* At least 50% decrease from baseline in MADRS score

*Design:* double blind RCT, ITT analysis, LOCF approach.

*Results:* 38 patients in the paroxetine group (43.7%) and 33 patients in the amitriptyline group (37.8%) were responders (p= 0.441). 47 patients in the first and 53 from the second group reported at least one adverse experience. There were 16 withdrawals in the paroxetine group and 19 withdrawals in the amitriptyline group. The study showed that paroxetine and amitriptyline were similar in efficacy and tolerability.

Cancurtaran et al., 2008

*Number of patients:* 53 patients participated in this study: 20 patients in the mirtazapine group, 13 patients in the imipramine group and 20 patients in a control group without medication or placebo (only supportive psychotherapy).

*Type of cancer/Stages:* The type and stage of cancer are not further specified.

*Duration:* six seeks

*Evaluation tools:* The patients were screened with the Structured Clinical Interview for Diagnostic (SCID). The Hospital Anxiety and Depression Scale (HADS) was administered for assessment of depression and anxiety during the study.

*Inclusion criteria:* Major depressive disorder, adjustment disorder, anxiety disorders.

*Response criteria:* The authors reported on the statistical significance of the rating differences between baseline and endpoint in each group.

*Design:* double blind RCT (not blinded for those who denied medication and received only psychotherapy), completers’ analysis.

*Results:* The patients in the mirtazapine group improved significantly after the six weeks in the total HADS scores (p=0.014), the anxiety subscale (p=0.04) and the depression subscale (p=0.008). The patients in the other two groups did not show any significant improvement.

B. **Placebo controlled trials**

The pharmacological agents that were used in these six studies were: mianserin (two trials), fluoxetine (three trials) and paroxetine (one trial). Musselman et al. used an additional third group with cancer patients receiving desipramine.

*Mianserin*

Costa 1985 and van Heeringen 1996 compared mianserin with placebo. Both reported a significant improvement of the depressive symptoms. Mianserin is a tetracyclic antidepressant agent and is considered as the predecessor of mirtazapine.

Costa et al., 1985

*Number of patients:* 73 patients participated in this study (36 in the drug group and 37 in the placebo group).

*Type of cancer/Stages:* Gynecological cancers, stages II, III, IV

*Duration:* four weeks

*Screening/Evaluation tools:* Zung Self-Rating Depression Scale (ZSRDS), Hamilton Depression Rating Scale. Secondary tools measures: Clinical Global Impression of Illness Severity (CGI-S).

*Inclusion Criteria:* ZSRDS over 41 and HDRS score over 16.

*Response criteria:* nor reported.

*Design:* Double blind RCT, ITT analysis, LOCF approach.

*Results:* 28 of the 36 patients of the mianserin group were responders. There were only 18 responders among the 37 participants in the placebo group (response evaluated according to the CGI scores). This difference was statically significant (P<0.025, Chi squared= 6.62). The primary outcome was based on the scores of HDRS. There were 7 dropouts in the mianserin group and 15 in the placebo group. 17 patients from the drug group and 11 from the placebo group reported side effects. This difference was not statistically significant (Chi squared = 2.11).

Van Heeringen and Zivkov, 1996

*Number of patients:* 55 participants in the study (28 in the mianserin group and 27 in the placebo group)

*Type of cancer/Stages:* Breast cancer. Stages I, II

*Duration:* 7 weeks

*Screening tool:* Hamilton Depression Rating Scale (HDRS)

*Inclusion Criteria:* HDRS score over 16

*Response criteria:* 50% decrease in baseline HRDS scores

*Design:* double blind RCT, ITT analysis, LOCF approach.

*Results:* 19 patients from the 28 ones in the mianserin group and 10 patients from the 27 ones in the placebo group were responders. This difference was statistically significant (p=0.044, Fisher’s exact test). Significantly more placebo treated patients (n=15) than mianserin treated patients (n=6) terminated prematurely the study (P=0.014, Fisher’s exact test). No significant differences were found between numbers of patients complaining of at least one adverse effect (AE) at any assessment point. At day 42, 11 mianserin- and 17 placebo- treated patients reported AEs (P=0.14).

*Fluoxetine*

Three RTCs compared fluoxetine with placebo.

Razavi et al., 1996

*Number of patients:* 91 patients participated in this study (45 in the fluoxetine group and 46 in the placebo group)

*Type of cancer/Stages:* Any kind of cancer (mainly gynecological), any stage (95% without metastases).

*Duration:* five weeks

*Screening tool:* Hospital Anxiety and Depression Scale (HADS). Other measures: the Montgomery and Asberg Depression Scale (MADRS), the Hamilton Anxiety Scale (HAS), the revised Symptom Checklist (SCL-90R) and the Spitzer Quality of Life Index (SQOLI).

*Inclusion Criteria:* a HADS score over 13

*Response criteria:* a 50% decrease or more in the baseline HADS scores

*Design:* double blind RCT, completers’ analysis.

*Results:* The HADS response rates were 18% in the fluoxetine group and 20% in the placebo group. The success rates (HADS scores under 8 at the end of the trial) were 11% (5 patients) and 7% (3 patients) in the drug and placebo group respectively. As a secondary outcome the author reported the response rates in both groups according to MADRS scale (50% improvement): these were 31% (14 patients) for the fluoxetine group and 33% (15 patients) in the placebo group. These differences were not statistically significant. Significantly more drop outs were observed in the drug group (n=15) than in the placebo group (n=7) (Chi Square test; P=0.04). 67% from the fluoxetine group reported at least one AE compared to 59% in the placebo group (not statistically significant difference: P= 0.43).

Fisch et al., 2003

*Number of patients:* 163 patients participated in the study (83 patients in the fluoxetine group and 80 patients in the placebo group).

*Type of cancer/Stages:* Any type of cancer in advanced stage.

*Duration:* twelve weeks

*Screening tool:* The Two Question Screening Survey (TQSS) was used to assess mood and anhedonia. The two questions are: “During the past month have you been bothered by feeling down, depressed or hopeless?” and “During the past month have you been bothered by having little interest or pleasure in doing things?” There are five possible answers which are assigned values from 0 to 4:

0 not at all, 1 a little bit, 2 somewhat, 3 quite a bit, 4 very much.

For the evaluation of depression and quality of life in the participants the following tools were used: the Functional Assessment of Cancer Therapy General instrument (FACT-G), the Functional Assessment of Chronic Illness Therapy Spiritual and the Brief Zung Self administered Depression Rating Scale (BZDRS) with 11 items.

*Inclusion Criteria:* A TQSS score of 2 or greater. Patients with a major depressive episode were excluded.

*Response criteria:* A best change score of at least -3 in the BZDRS. A best change score is defined as the difference between the baseline score and the average of the best consecutive scores.

*Design:* Double blind RCT. Computations were made only on patients who completed the baseline questionnaires (n=159) and at least one follow up assessment (n=129). The authors used additionally the generalized estimating equation (GEE) method of regression.

*Results:* There were data for 129 patients at the end point (64 in the fluoxetine group and 65 in the placebo group). 48% (n=31) patients in the fluoxetine group and 36% (n=23) in the placebo group were responders. This difference was not statistically significant. Reevaluating the data with the GEE method of regression the authors found a significant improvement in the fluoxetine group. There were not any concrete data about the number of dropouts or reported adverse effects in each group.

Navari et al., 2008

*Number of patients:* 203 patients out of the 357 screened patients qualified for enrolment in this study. From the 193 who enrolled in the study the authors reported on the 180 patients who completed the six month assessment.

*Type of cancer/Stages:* Breast cancer, stages I, II

*Duration:* six months

*Screening tool:* The Two Question Screening Survey (TQSS) was used to assess mood and anhedonia. The two questions are: “During the past month have you been bothered by feeling down, depressed or hopeless?” and “During the past month have you been bothered by having little interest or pleasure in doing things?” There five possible answers which are assigned values from 0 to 4:

0 not at all, 1 a little bit, 2 somewhat, 3 quite a bit, 4 very much.

The quality of life was estimated using the Functional Assessment of Cancer Therapy-General (FACT-G) and the depression was estimated with the Brief Zung self administered Depression Rating Scale (BZDRS).

*Inclusion Criteria:* A TQSS score of 2 or greater. Patients with a major depressive episode were excluded.

*Response criteria:* “significant improvement” in the BZSRS (not further defined).

*Design:* double blind RCT, completers’ analysis.

*Results:* 71 patients from the fluoxetine group and 23 patients from the placebo group had a significant improvement in depressive symptoms (P<0.0005). There were not any data about adverse effects.

*Paroxetine*

One study compared paroxetine with desipramine and placebo in depressed cancer patients.

Musselman et al., 2006

*Number of patients:* 35 patients participated in this study. They were divided into three groups and assigned to receive either paroxetine (n=13) or desipramine (n=11) or placebo (n=11).

*Type of cancer/Stages:* Breast cancer at any stage.

*Duration:* six weeks

*Screening tool:* a DSM-III-R multi-axial evaluation. Other rating scales used were the 21-item Hamilton Rating Scale for Depression (HAM-D), the 14-item Hamilton Rating Scale for Anxiety and the Clinical Global Impression Scale (CGI).

*Inclusion Criteria:* DSM-III-R criteria for major depression (except duration of illness had to be at least on month), HAM-D score of at least 14 in the first 17 items of the 21-item HAM-D.

*Response criteria:* A decrease of ≥50% from baseline HAM-D score or a CGI global improvement score ≤-2. Clinical remission was defined as a HAM-D score ≤7.

*Design:* double blind RCT, ITT analysis, LOCF approach

*Results:* The response rates were: 38% (5/13 patients) in the paroxetine group, 45% (5/11 patients) in the desipramine group and 55% (6/11 patients) in the placebo group (p= 0.91). The remission rates were: 23% (3/11 patients) in the paroxetine group, 45% (5/11 patients) in the desipramine group and 36% (4/11 patients) in the placebo group (p=0.55). There were 14 dropouts by week six: 5 in the paroxetine group, 4 in the desipramine group and 5 in the placebo group.

The most common adverse effects were:

Paroxetine: dry mouth (n=6), nausea (n=5), pain (n=5)

Desipramine: dry mouth (n=8), constipation (n=4), headaches (n=4), pain (n=4)

Placebo: headache (n=5), pain (n=5), dry mouth (n=3), constipation (n=3).

The difference in side effects among the groups was not significant.

### Appendix B

#### Assessment of bias

We used the Cochrane Collaboration’s tool for assessing the risk of bias. These criteria may be considered sufficiently strict. This included extracting of six domains and judging them. The consensual authors’ judgment were either “Yes”, indicating low risk of bias, “No” indicating high risk of bias, or “Unclear” indicating unknown risk of bias. The criteria to assess the studies were: (Tables 5, 6, 7, 8, 9, 10, 11, 12, 13, 14).

**Table 5:** Criteria to assess the risk for bias

**Domain**	**Description**	**Review author’s judgement**
Sequence generation	Describe the method used to generate the allocation sequence	Was the allocation sequence adequately generated? (Yes, No, Unclear)
Allocation concealment	Describe the method used to conceal the allocation sequence	Was allocation adequately concealed? (Yes, No, Unclear)
Blinding of participants, personnel, and outcome	Describe all measures used to blind participants and personnel	Was knowledge of the allocated intervention adequately prevented during the study? (Yes, No, Unclear)
Incomplete outcome data	Describe the completeness of outcome data for each main outcome including attrition and exclusions from the analysis.	Were incomplete outcome data adequately addressed? (Yes, No, Unclear)
Selective outcome reporting	State how the possibility of selective outcome reporting was examined by the review authors and what was found.	Are reports of the study free of suggestion of selective outcome reporting? (Yes, No, Unclear)
Other sources of bias	State any important concerns about bias not addressed in the other domains.	Was the study apparently free of other problems that could put it at high risk of bias?

Study-wise evaluation of Risk of Bias:

**Table 6:** Pezella et al., 2001

**Domain**	**Description**	**Review author’s judgement**
Sequence generation	Randomized study, method not described.	“Unclear”
Allocation concealment	Double dummy technique was used. Assignment envelopes are not described.	“Unclear”
Blinding of participants, personnel, and outcome	Double blind study. More frequent anticholinegic AEs in the amitriptyline group, which could break blinding.	“Unclear”
Incomplete outcome data	Main outcome was the MADRS score. 16 withdrawals in the paroxetine group and 19 in the amitriptyline group. 9 dropouts in the paroxetine group and 10 in the amitriptyline group due to AEs. Other reasons for withdrawals are not reported. Analysis according to ITT principle. Missing data imputation method: LOCF.	“No”
Selective outcome reporting	Protocol is not available. All pre-specified outcomes of interest are reported in the pre-specified way.	“Yes”
Other sources of bias	The study appears to be free of other sources of bias.	“Yes”

**Table 7:** Holland et al., 1998

**Domain**	**Description**	**Review author’s judgement**
Sequence generation	Randomized study, randomization method not described.	“Unclear”
Allocation concealment	Method is not described.	“Unclear”
Blinding of participants, personnel, and outcome	Double blind study. More frequent anticholinergic effects in the fluoxetine group. Issue is not sufficiently addressed by the authors.	“Unclear”
Incomplete outcome data	The main outcome war the raw baseline to endpoint differences in HAM-D. There were 6 dropouts in the fluoxetine group, all due to AEs. There were 7 dropouts in the desipramine, 4 due to AEs, 3 to unknown reasons. Analysis according to ITT principle. Missing data imputation method: LOCF.	“No”
Selective outcome reporting	Study protocol is not available. All pre-specified outcomes are reported in the pre-specified way. No response criteria were defined. Improvement was not pre-specified.	“No”
Other sources of bias	The study seems to be free from other sources of bias.	“Yes”

**Table 8:** Cancurtaran et al., 2008

**Domain**	**Description**	**Review author’s judgement**
Sequence generation	Randomization method is not described. Patients who refused to take medication formed a control group. The allocation by preference of the participants is problematic in randomized studies.	“No”
Allocation concealment	Not described for the two drug groups. No blinding for the control group.	“No”
Blinding of participants, personnel, and outcome	No blinding for the control group.	“No”
Incomplete outcome data	The main outcome was the HAM-D score and single symptom scales score for nausea, pain, vomiting. 4 Dropouts in the mirtazapine group, 4 in the imipramine group, ten dropouts in the control group. No ITT analysis. Missing data imputation method: completers’ analysis.	“No”
Selective outcome reporting	No study protocol available. All pre-specified outcomes are reported in the pre-specified way. No pre-specified criteria for response or improvement.	“No”
Other sources of bias	The study seems to be free from other sources of bias.	“Yes”

**Table 9:** Costa et al., 1985

**Domain**	**Description**	**Review author’s judgement**
Sequence generation	The randomization method is not described.	“Unclear”
Allocation concealment	Exact method is not described.	“Unclear”
Blinding of participants, personnel, and outcome	Double blind trial. The issue is not adequately addressed.	“Unclear”
Incomplete outcome data	The main outcome was the HDRS score. 7 dropout in the mianserin group (MG) and 15 in the placebo group (PG). Reasons for withdrawal: 1 in each group due to AEs, 2 in PG due to lack of efficacy, the treatment by one patient in the PG was interrupted by the investigator, 2 in MG and 4 MG ended the anticancer treatment, 2 in MG due to cancer complications, 1 in MG and 2 in PG due to temporary withdrawal from the anticancer treatment, 2 in PG refused anticancer therapy and were dismissed, 3 in PG had problems at home and 1 in MG died. The authors used an ITT analysis. Missing data imputation method: LOCF approach. Proportionally about one third (30%) of the patients were dropouts, which can induce bias in intervention effect estimate.	“No”
Selective outcome reporting	No study protocol available. All pre-specified outcomes are reported in the pre-specified way. The response criteria are not pre-specified.	“No”
Other sources of bias	The study seems to be free of other sources of bias.	“Yes”

**Table 10:** Van Heeringen et al., 1996

**Domain**	**Description**	**Review author’s judgement**
Sequence generation	The randomization method is not described	“Unclear”
Allocation concealment	The exact method is not described.	“Unclear”
Blinding of participants, personnel, and outcome	The study is defined as double blind. The issue is not addressed by the authors.	“Unclear”
Incomplete outcome data	The main outcome was the HDRS score. There were 6 drop outs in the mianserin group and 15 dropouts in the placebo group. 2 patients in the mianserin group and 11 in the placebo group withdrew due to lack of efficacy. 2 dropouts in the mianserin group and 4 in the placebo group due to AEs. ITT analysis. Missing data imputation method: LOCF approach. Over one third of the patients withdrew from the study (38%), No study protocol available. All pre-specified outcomes are reported in the pre-specified way.	“No”
Selective outcome reporting	No study protocol available. All pre-specified outcomes are reported in the pre-specified way.	“Yes”
Other sources of bias	The study seems to be free of other sources of bias.	“Yes”

**Table 11:** Razavi et al., 1996

**Domain**	**Description**	**Review author’s judgement**
Sequence generation	Randomization method is not described.	“Unclear”
Allocation concealment	The exact method is not described.	“Unclear”
Blinding of participants, personnel, and outcome	Double blind trial. The authors do not address this issue.	“Unclear”
Incomplete outcome data	The main outcome was the number of patients with success criteria (HADS score≤8) and with response criteria (≥50% improvement in HADS score). There were 15 dropouts in the fluoxetine group (FG) and 7 dropouts in the placebo group (PG). The reasons for dropouts in the FG were: 7 due to AEs, 3 decided to interrupt their participation for unknown reasons, 1 due to alcohol abuse, and 4 for other reasons:	“Unclear”
	(Non-compliance, investigator’s decision, lost to follow-up). The reasons for dropouts in the PG were: 2 due to concomitant events, 4 for unknown reasons, 1 for psychiatric reasons. The authors used an ITT basis for the success and response rates. The exact missing data imputation method is not being reported.	
Selective outcome reporting	No study protocol available. All pre-specified outcomes are reported in the pre-specified way.	“Yes”
Other sources of bias	The study seems to be free of other sources of bias.	“Yes”

**Table 12:** Fisch et al., 2003

**Domain**	**Description**	**Review author’s judgement**
Sequence generation	Randomization by means of a preprinted randomization table.	“Yes”
Allocation concealment	The exact method is not described.	“Unclear”
Blinding of participants, personnel, and outcome	“The issue is not addressed by the authors.	“Unclear”
Incomplete outcome data	163 patients were randomized and 159 allocated to receive medication. The patients were included in the analysis if they provided data at least two assessments (baseline and one of the next four). 64 patients were evaluable in the fluoxetine group and 65 in the placebo group. The reasons for dropouts are not fully presented. The authors used a modification of completers’ analysis. The missing data imputation method was the best change score, which is defined as the difference between the baseline score and the average of the best consecutive scores. According to the authors’ opinion this is a valid statistical method for longitudinal data. To our opinion the best change score belongs to the inappropriate imputation methods.	“No”
Selective outcome reporting	No study protocol available. All pre-specified outcomes are reported in the pre-specified way.	“Yes”
Other sources of bias	There were many loss of data especially at the fourth assessment. This could influence the intervention effect estimate.	“No”

**Table 13:** Navari et al., 2007

**Domain**	**Description**	**Review author’s judgement**
Sequence generation	The randomization method is not described.	“Unclear”
Allocation concealment	The exact method is not described.	“Unclear”
Blinding of participants, personnel, and outcome	Double blind trial. The issue is not addressed by the authors.	“Unclear”
Incomplete outcome data	The main outcome was the scores on FACT-G and BZSDS. 193 patients enrolled in the study, 183 were available at the first follow-up and 180 at the second. The reasons for dropouts are not reported. The authors used a completers’ analysis, which was not pre-specified in the description of the study.	“No”
Selective outcome reporting	The scores of the FACT-G and BZSDS are not reported. The results are presented as numbers of patients with significant improvement, which is not pre-specified in the description of the study. The AEs are also not reported.	“No”
Other sources of bias	The study seems to be free from other sources of bias.	“Yes”

**Table 14:** Musselmann et al., 2006

**Domain**	**Description**	**Review author’s judgement**
Sequence generation	The randomization method is not described	“Unclear”
Allocation concealment	The exact method is not described.	“Unclear”
Blinding of participants, personnel, and outcome	Double blind study. The issue is not addressed by the authors.	“Unclear”
Incomplete outcome data	The main outcome was the number of patients with response (≥50% improvement in the HAM-D scale) and with remission (HAM-D≤7). There were 14 dropouts in a total of 40 participants (40%). Reasons for dropouts were: AEs (2 in paroxetine group, 1 in desipramine group and 2 in placebo group), lack of efficacy (2 in paroxetine and 2 in placebo group), patients’ wish to discontinue (2 in placebo group), one was lost to follow-up and one from the placebo group could not ingest any medication. The analysis was done on an ITT base. The missing data imputation method was the LOCF.	“No”
Selective outcome reporting	No study protocol available. All pre-specified outcomes are reported in the pre-specified way.	“Yes”
Other sources of bias	Small number of participants.	“No”

**Figure 5:**
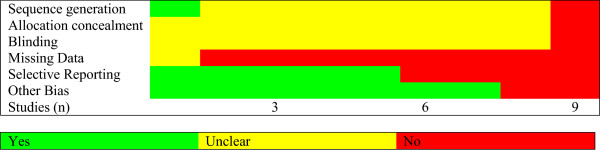
**Risk of bias graph for all 9 studies reviewed. **Most studies did not describe the methods used to generate and conceal the allocation sequence. All studies were defined as double blind, but the exact blinding method was not described in any of them. No study used an appropriate method to address the issue of missing data. As one can see the studies were relative uniform as far as the issue of risk of bias is concerned.

In summary, only one study (Fisch et al., 2003) described the exact randomization method. The rest studies defined themselves as randomized, but did not describe the method (“Unclear”). The exact allocation concealment method was not described in any study. All studies were defined as double blind. No article reported which persons in the study were blinded and which were not. All but one studies described an inappropriate missing data imputation method, even the ones who used an ITT-based analysis; the LOCF method can also introduce bias (see Cochrane [31] and http://www.missingdata.org.uk). In total several indications for Risk of Bias can be observed and most items remained unclear (see Figures 3 and 5).

### Appendix C

#### Subgroup analysis after outlier exclusion

1. Eligibility criteria: Depression vs. depressive symptoms

Subgroup analysis for inclusion criteria (Table 15).

**Table 15:** Subgroup analysis for inclusion criteria

**Author**	**Year**	**Verum**	**Verum**		**Placebo**	
			**Participants**	**Responders**	**Participants**	**Responders**
**Group A: Clinical depression as an inclusion criterion**						
Costa	1985	Mianserin	36	28	37	18
Van Heeringen	1996	Mianserin	28	19	27	10
Razavi	1996	Fluoxetine	35	8	46	9
Musselmann	2006	Paroxetine/desipramine	24	11	11	6
*Total*			133	66	121	43
**Group B: Clinical depression not as an inclusion criterion**						
Fisch	2003	Fluoxetine	64	31	65	23
*Total*			64	31	65	23

RR_(A)_= 1.42, RR_(B)_= 1.37 (Z_diff_= 0.19, p=0.85).

2. Study design: Intention to treat vs. completers’ analysis

Subgroup analysis for study design (ITT vs. completers’ analysis) (Table 16)

**Table 16:** Subgroup analysis for study design (ITT vs. completers’ analysis)

**Author**	**Year**	**Drug**	**Verum**		**Placebo**	
			**Participants**	**Responders**	**Participants**	**Responders**
**Group A: Analyses by ITT**						
Costa	1985	Mianserin	36	28	37	18
Van Heeringen	1996	Mianserin	28	19	27	10
Musselmann	2006	Paroxetine/desipramine	24	11	11	6
*Total*			88	58	75	34
**Group B: analyses not by ITT**						
Fisch	2003	Fluoxetine	64	31	65	23
Razavi	1996	Fluoxetine	45	8	46	9
*Total*			109	39	111	32

ITT: intention to treat.

RR_(A)_ = 1.49, RR _(B)_ = 1.27 (Z_diff_= 0.93, p= 0.36).

3. Eligibility criteria: Cancer stage

Subgroup analysis for cancer stage (Table 17).

**Table 17:** Subgroup analysis for cancer stage

**Author**	**Year**	**Drug**	**Verum**		**Placebo**	
			**Participants**	**Responders**	**Participants**	**Responders**
**Group A: Any or advanced cancer stage**						
Costa	1985	Mianserin	36	28	37	18
Fisch	2003	Fluoxetine	64	31	65	23
Razavi	1996	Fluoxetine	45	8	46	9
Musselmann	2006	Paroxetine/desipramine	24	11	11	6
*Total*			169	78	159	56
**Group B: Early cancer stages**						
Van Heeringen	1996	Mianserin	28	19	27	10
*Total*			28	19	27	10

RR_(A)_= 1.29, RR_(B)_= 1.83 (Z_diff_= - 1.73, p=0.11).

4. Comparison between SSRIs and tetracyclic antidepressants

Subgroup analysis for type of antidepressant (Table 18)

**Table 18:** Subgroup analysis for type of antidepressant

**Author**	**Year**	**Drug**	**Verum**		**Placebo**	
			**Participants**	**Responders**	**Participants**	**Responders**
**Group A: SSRIs**						
Fisch	2003	Fluoxetine	64	31	65	23
Razavi	1996	Fluoxetine	45	8	46	9
Musselmann	2006	Paroxetine	13	5	11	6
*Total*			122	44	122	38
**Group B: Mianserin**						
Costa	1985	Mianserin	36	28	37	18
Van Heeringen	1996	Mianserin	28	19	27	10
*Total*			64	47	64	28

RR_(A)_=1.16, RR_(B)_= 1.67 (Z_diff_= -2.17, p= 0.03).

### Appendix D

List of the final 38 studies (Table 19)

**Table 19:** List of the final 38 studies

**Article**	**Assessment**
Goldberg RJ. Management of depression in the patient with advanced cancer. JAMA.246(4):373–6, 1981.	Review
Costa D, Mogos I, Toma T. Efficacy of mianserin in the treatment of depression of women with cancer. Acta Psychiatrica Scandinavica. 72 (suppl. 320): 85–92, 1985.	**RCT included in the review**
Mathé G, Lopez MD, Fréchet M, de Vassal F, Brienza S, Gastiaburu J. A comparative trial of a MAOI, iproniazide, and a polycyclic agent, mianserine, for the search of the most rapidly and frequently active treatment of depressive syndromes in an oncology service. Biomedicine and Pharmacotherapy.41(1):13–26, 1987.	No double blind RCT
Maguire P, Hopwood P, Tarrier N, Howell T. Treatment of depression in cancer patients. Acta Psychiatrica Scandinavica Suppl. 320:81–4, 1985.	Antidepressant therapy was administrated together with anxiolytic and supportive psychotherapy
Evans DL, McCartney CF, Nemeroff CB, Haggerty JJ Jr, Simon JS, Pedersen CA, Holmes V, Droba M, Mason GA, Raft D, et al. Depression in cancer patients. Diagnostic and treatment considerations. North Carolina Medical Journal.49(10):546–8, 1988.	Review
Silberfarb PM. Psychiatric treatment of the patient during cancer therapy. CA; A Cancer Journal of Clinicians. 38(3):133–7, 1988.	Review
Evans DL, McCartney CF, Haggerty JJ Jr, Nemeroff CB, Golden RN, Simon JB, Quade D, Holmes V, Droba M, Mason GA, et al. Treatment of depression in cancer patients is associated with better life adaptation: a pilot study. Psychosomatic Medicine. 50(1):73–6, 1988.	No control group.
Van Heeringen K, Zivkov M. Pharmacological treatment of depression in cancer patients. A placebo controlled study of Mianserin. British Journal of Psychiatry. 169: 440.443, 1996.	**RCT included in the review**
Razavi D, Allilaire JF, Smith M, Salimpour A., Verra M, Desclaux B, Saltel P, Piollet I, Gauvain-Piquard A., Trichard C, Cordier B, Fresco R, Guillibert E, Sechter D, Orth JP, Bouhassira M, Mesters P, Blin P. The effect of fluoxetine on anxiety and depression symptoms in cancer patients. Acta Psychiatrica Scandinavia. 94: 205–210, 1996.	**RCT included in the review**
Holland JC, Romano SJ, Heiligenstein JH, Tepner RG, Wilson MG. A controlled trial of fluoxetine and desipramine in depressed women with advanced cancer. Psycho-Oncology. 7: 291–200, 1998	**RCT included in the review**
Razavi D, Kormoss N, Collard A, Farvacques C, Delvaux N. Comparative study of the efficacy and safety of trazodone versus clorazepate in the treatment of adjustment disorders in cancer patients: a pilot study. The Journal of International Medical Research. 27(6):264–72, 1999.	The efficacy of trazodone cannot be safely proven when it is compared to a benzodiazepine. Depression was not an eligibility criterion.
Musselmann DL, Lawson DH, Gumnick JF, Manatunga AK, Penna S, Goodkin RS, Greiner K, Nemeroff CB, Miller AH. Paroxetine for the prevention of depression induced by high dose interferone alpha. The New England Journal of Medicine. Vol 344, No 13, 2001	Prevention study, thus prevention was not an eligibility criterion.
Pezella G, Moslinger-Gehmayr R, Contu A. Treatment of depression in patients with breast cancer: a comparison between paroxetine and amitrptyline. Breast Cancer Research and Treatment. 70: 1–10, 2001	**RCT included in the review**
Passik SD, Kirsh KL, Theobald D, Donaghy K, Holtsclaw E, Edgerton S, Dugan W. Use of a depression screening tool and a fluoxetine-based algorithm to improve the recognition and treatment of depression in cancer patients. A demonstration project. Journal of Pain and Symptom Management. 24(3):318–27, 2002.	No RCT.
Carr D, Goudas L, Lawrence D, Pirl W, Lau J, DeVine D, Kupelnick B, Miller K. Management of cancer symptoms: pain, depression, and fatigue. Evidence Report/Technology Assessment. 61:1–5, 2002.	Review.
Davis MP, Khawam E, Pozuelo L, Lagman R. Management of symptoms associated with advanced cancer: olanzapine and mirtazapine. A World Health Organization project. **Expert Review of Anticancer Therapy. 2(4): 365–76, 2002.**	Recommendation
Fisch MJ, Loehrer PJ, Kristeller J, Passik S, Jung SH, Shen SH, Arquette MA, Brames MJ, Einhorn LH. Fluoxetine versus Placebo in advanced cancer outpatients: a double-blinded trial of the Hoosier oncology group. Journal of Clinical Oncology. Vol 21, No 10: 1937–1943, 2003.	**RCT included in the review**
Theobald DE, Kirsh KL, Holtsclaw E, Donaghy K, Passik SD. An open label pilot study of citalopram for depression and boredom in ambulatory cancer patients. **Palliat Support Care. 2003 Mar;1(1):71–7.**	No RCT.
Morrow GR, Hickok JT, Roscoe JA, Raubertas RF, Andrews PLR, Flynn PJ, Hynes HE, Banerjee TK, Kirschner JJ, King DK. Differential effects of paroxetine on fatigue and depression: a randomized, double blind trial from the University of Rochester Cancer Center Community Clinical Oncology Program. Journal of Clinical Oncology. Vol 21, No 24: 4635–4641, 2003	Depression was not an eligibility criterion
Pae CU, Kim YJ, Won WY, Kim HJ, Lee S, Lee CU, Lee SJ, Kim DW, Lee C, Min WS, Kim CC, Paik IH, Serretti A. Paroxetine in the treatment of depressed patients with haematological malignancy: an open-label study. Human Psychopharmacology. 19(1):25–9, 2004.	No RCT.
Coyne JC, Palmer SC, Shapiro PJ. Prescribing antidepressants to advanced cancer patients with mild depressive symptoms is not justified. Journal of Clinical Oncology. 1;22(1):205–6; author reply 206–8, 2004.	Comment.
Thangathurai D, Roffey P, Mogos M, Riad M, Mikhail M. Usefulness of desipramine in ICU cancer patients for acute depression. Journal of Palliative Care. 20(4):326, 2004.	Comment.
Ladd CO, Newport DJ, Ragan KA, Loughhead A, Stowe ZN. Venlafaxine in the treatment of depressive and vasomotor symptoms in women with perimenopausal depression. Depression and Anxiety. 22(2):94–7, 2005.	No RCT.
Roscoe JA, Morrow GR, Hickok JT, Mustian KM, Griggs JJ, Matteson SE, Bushunow P, Qazo R, Smith B. Effect of paroxetine hydrochloride on fatigue and depression in breast cancer patients receiving chemotherapy. Breast Cancer Research and Treatment. 89: 243–249, 2005.	Depression was not an eligibility criterion.
Musselmann DL, Somerset WI, Guo Y, Manatunga AK, Porter M, Penna S, Lewison B, Goodkin R, Lawson K, Lawson D, Evans DL, Nemeroff CB. A double-blind multicenter parallel-group study of paroxetine, desipramine or placebo in breast cancer patients (stages I, II, III, IV) with major depression. Journal of Clinical Psychiatry. 67: 288–296, 2006.	**RCT included in the review.**
Kimmick GG, Lovato J, McQuellon R, Robinson E, Muss HB. Randomized, double-blind, placebo-controlled, crossover study of sertraline (Zoloft) for the treatment of hot flashes in women with early stage breast cancer taking tamoxifen. **The Breast Journal. 12(2):114–22, 2006.**	Depression was a secondary outcome.
Moss EL, Simpson JS, Pelletier G, Forsyth P. An open-label study of the effects of bupropion SR on fatigue, depression and quality of life of mixed-site cancer patients and their partners. Psychooncology. 15(3):259–67, 2006.	No RCT.
Loibl S, Schwedler K, von Minckwitz G, Strohmeier R, Mehta KM, Kaufmann M. Venlafaxine is superior to clonidine as treatment of hot flashes in breast cancer patients--a double-blind, randomized study. **Annals of Oncology. 18(4):689–93, 2007.**	No measures for depression were included.
Stockler MR, O´Connel R, Nowak AK, Goldstein D, Turner J, Wilcken NRC, Wyld D, Abdi EA, Glasgow A, Beale PJ, Jefford M, Dhillon H, Heritier S, Carter C, Hickie IB, Simes RJ. Effect of sertraline on symptoms and survival in patients with advanced cancer, but without major depression: a placebo controlled double-blind randomized trial. Lancet Oncology. 8: 603–612, 2007.	Depression was not an eligibility criterion.
Raji MA, Barnum PD, Freeman J, Markowitz AB. Mirtazapine for depression and comorbidities in older patients with cancer. **Annals of Pharmacotherapy.41(9):1548–9, 2007.**	No RCT.
Cankurtaran ES, Ozalp E, Soygur H, Akbiyik DI, Tuhan L, Alkis N. Mirtazapine improves sleep and lowers anxiety and depression in cancer patients: superiority over imipramine. Supportive Care in Cancer. 16: 1291–1298, 2008.	**RCT included in the review.**
Torta R, Siri I, Caldera P. Sertraline effectiveness and safety in depressed oncological patients. Supportive Care in Cancer. 16(1):83–91, 2008.	No RCT.
Okamura M, Akizuki N, Nakano T, Shimizu K, Ito T, Akechi T, Uchitomi Y. Clinical experience of the use of a pharmacological treatment algorithm for major depressive disorder in patients with advanced cancer. **Psychooncology. 17(2):154–60, 2008.**	No RCT.
Ersoy MA, Noyan AM, Elbi H. An open-label long-term naturalistic study of mirtazapine treatment for depression in cancer patients. **Clinical Drug Investigation. 28(2):113–20, 2008.**	No RCT.
Kim SW, Shin IS, Kim JM, Kim YC, Kim KS, Kim KM, Yang SJ, Yoon JS. Effectiveness of mirtazapine for nausea and insomnia in cancer patients with depression. **Psychiatry and Clinical Neuroscience. 62(1):75–83, 2008.**	No RCT.
Lydiatt WM, Denman D, McNeilly DP, Puumula SE, Burke WJ. A randomized placebo- controlled trial of citalopram for the prevention of major depression during treatment for head and neck cancer. Archives of Otolaryngology- Head and Neck Surgery. Vol. 134 (No 5), 2008.	Prevention study, thus depression was not an eligibility criterion.
Navari RM, Brenner MC, Wilson MN. Treatment of depressive symptoms in patients with early stage breast cancer undergoing adjuvant therapy. Breast Cancer Research and Treatment. 112: 197–201, 2008.	RCT included in the review.
Thangathurai D, Roby J, Roffey P. Treatment of resistant depression in patients with cancer with low doses of ketamine and desipramine. Journal of Palliative Medicine. 13(3):235, 2010.	The authors report on two patients

## Competing interests

The authors declare that they have no competing interests.

## Authors’ contributions

Zacharias G. Laoutidis participated in data collection and evaluation, performed the statistical analysis and drafted the manuscript. Klaus Mathiak conceived and designed the study, acquired the funding, participated in and supervised collection of data, and helped to draft the manuscript. Both authors read and approved the manuscript.

## Pre-publication history

The pre-publication history for this paper can be accessed here:

http://www.biomedcentral.com/1471-244X/13/140/prepub
